# Carcinogenic effect of human tumor-derived cell-free filtrates in nude mice

**DOI:** 10.3389/fmolb.2024.1361377

**Published:** 2024-04-18

**Authors:** Jorge Berlanga-Acosta, Ernesto Arteaga-Hernandez, Ariana Garcia-Ojalvo, Dayanis Duvergel-Calderin, Marisol Rodriguez-Touseiro, Laura Lopez-Marin, Jose Suarez-Alba, Dasha Fuentes-Morales, Osmany Mendoza-Fuentes, Sheyla Fernández-Puentes, Yanier Nuñez-Figueredo, Gerardo Guillen-Nieto

**Affiliations:** ^1^ Center for Genetic Engineering and Biotechnology, Biomedical Research Direction, Havana, Cuba; ^2^ Department of Pathology, Hermanos Ameijeiras Hospital, La Habana, Cuba; ^3^ Department of Pathology, Institute for Arteriosclerosis Research, Institute of Nephrology “Dr. Abelardo Buch”, Havana, Cuba; ^4^ National Center for Laboratory Animal Breeding, Boyeros, Cuba; ^5^ Drug Research and Development Center (CIDEM), La Habana, Cuba

**Keywords:** cells-free filtrate, tumor homogenate, cancer, nude mice, malignant transformation

## Abstract

Cancer remains a worldwide cause of morbidity and mortality. Investigational research efforts have included the administration of tumor-derived extracts to healthy animals. Having previously demonstrated that the administration of non-transmissible, human cancer-derived homogenates induced malignant tumors in mice, here, we examined the consequences of administering 50 or 100 µg of protein of crude homogenates from mammary carcinoma, pancreatic adenocarcinoma, and melanoma samples in 6 inoculations per week during 2 months. The concurrent control mice received homogenates of healthy donor-skin cosmetic surgery fragments. Mammary carcinoma homogenate administration did not provoke the deterioration or mortality of the animals. Multiple foci of lung adenocarcinomas with a broad expression of malignity histomarkers coexisting with small cell-like carcinomas were found. Disseminated cells, positive to classic epithelial markers, were detected in lymphoid nodes. The administration of pancreatic tumor and melanoma homogenates progressively deteriorated animal health. Pancreatic tumor induced poorly differentiated lung adenocarcinomas and pancreatic islet hyperplasia. Melanoma affected lungs with solid pseudopapillary adenocarcinomas. Giant atypical hepatocytes were also observed. The kidney exhibited dispersed foci of neoplastic cells within a desmoplastic matrix. Nuclear overlapping with hyperchromatic nuclei, mitotic figures, and prominent nuclear atypia was identified in epidermal cells. None of these changes were ever detected in the control mice. Furthermore, the incubation of zebrafish embryos with breast tumor homogenates induced the expression of c-M*yc* and HER-2 as tumor markers, contrasting to embryos exposed to healthy tissue-derived material. This study confirms and extends our hypothesis that tumor homogenates contain and may act as vectors for “malignancy drivers,” which ultimately implement a carcinogenesis process in otherwise healthy mice.

## Highlights

• Here, we describe that homogenates prepared using fresh surgical samples of three types of human malignant tumors using mechanical disruption in sterile physiologic saline solution with no other type of chemical manipulation disrupt the proliferative and differentiation programs of normal host cells, leading to the onset of premalignant and malignant changes in otherwise healthy nude mice
• These neoplastic changes occurred in a narrow temporary window
• We also show that 60 h zebrafish embryos incubation with breast cancer cell-free filtrates (CFFs) elicited the expression of mature proteins of tumor markers as c-Myc and HER-2
• This malignant homogenate was also shown to morphologically and functionally impact the embryo homeostasis, suggesting that some signaler contained in the pathologic CFF “interacted” with the embryo cells
• This study confirms, extends, and supports our hypothesis that human malignant tissue-derived homogenates contain soluble drivers of donor cells that “enslave” host cells to recapitulate malignant histopathologic hallmarks in a relatively short period of time
• This study is also the fourth in a line that concurs to support the hypothetical existence and transmissibility of a “pathologic cellular memory,” encrypted in the “diseased cells,” with no interspecies barrier, and that it is able to impose the recapitulation of the pathologic traits of the human donor

## Introduction

The molecular drivers and pathways behind the multifaceted episode of cellular malignant transformation up to clinical cancer have remained elusive for years (Bordonaro, 2019; NCI, 2021). Thus, although no other human pathology has been so extensively and comprehensively investigated, the broad horizon of cancer mysteries still needs to be studied. Regardless of the large repertoire of innovative therapies available, this heterogeneous group of diseases still stands as a major cause of mortality worldwide (Siegel et al., 2023).

Carcinogenesis is a vast process that stems from genetic and epigenetic alterations, including loss- and gain-of-function mutations that occur in a single cell (Cataisson et al., 2022; Lai et al., 2023). This seems to be a sequence of cumulative changes from initiation, promotion, and progression (Nijman, 2020; Sarwar et al., 2023), and it translates to an expanding disorder in the cellular organization under micro-environmental pressure for survival (Nijman, 2020). Consequently, the transformed cells have exclusive and distinctive capabilities, including epigenetic plasticity, cytoprotective abilities, metabolic reprogramming, proliferative dynamism, dissemination skills, phenotypic transition, dormancy, and even local cannibalism (Kariagina et al., 2020; Missiroli et al., 2020; Phan and Croucher, 2020; Grunt and Heller, 2023; Torres et al., 2023).

The immortal and autonomous malignant cells also “educate” the host immune system with lessons of tolerance and, ironically, use (Hanahan, 2022; Grunt and Heller, 2023) inflammation for their own benefit (Gonzalez et al., 2018; Abbott and Ustoyev, 2019; Kim and Cho, 2022), which contributes to the process of metastatic seeding in the new substrate (Han et al., 2022; Weiss et al., 2022). Importantly, cancer cells are genuine “industries” of secreting soluble messages, being able to produce and deliver a broad variety of encapsulated or free signalers with multiple pathological implications (Pardini et al., 2019; Liu et al., 2023). After Bernard Peyrilhe inaugurated the investigative use of human cancer-derived fluids, the administration of filtered cell-free tumor homogenates to animals has translated into groundbreaking contributions to experimental pathology (Elemento, 2021). We recently undertook the use of cell-free filtrates (CFFs) derived from fresh human pathologic tissue samples to examine the hypothesis that the “chemical codes” of non-communicable diseases are imprinted in target tissues, which could be extracted, passively transferred to healthy animals, and accordingly reproduce the histological hallmarks of the human donor (Berlanga-Acosta et al., 2022a). These experiments demonstrated that CFFs acted as vehicles for delivering the soluble signalers that imposed the phenotypes of pathologic donors in otherwise normal animals (Berlanga-Acosta et al., 2021; Berlanga-Acosta et al., 2022b). Subsequently, and following the hypothesis that CFFs may contain cell-transforming messengers, we examined the effects of administering the homogenates of surgically excised malignant tumors in nude mice. Tumor-derived crude material induced premalignant and malignant changes in different organs in a narrow temporary window (Berlanga-Acosta et al., 2022c).

Here, we describe the findings of three independent and extemporaneous experiments consistent with that conducted by (1): the reproducibility of lung adenocarcinomas following the administration of CFFs derived from mammary ductal carcinomas (2), the induction of pre-neoplastic changes and truthful malignancies resulting from the administration of pancreatic adenocarcinoma and metastatic melanoma in otherwise normal nude mice, and (3) that zebrafish embryos exposed to breast carcinoma CFFs diluted in aquarium water broadly expressed HER-2 and overexpressed the C-M*yc* oncogene. All these lines of evidence confirm that CFFs contain soluble transforming messengers that may drive alterations of the proliferative and differentiation programs.

## Materials and methods

### Ethics and consent

The experimental protocols and the use of human tissues were reviewed and approved by the ethics committees of the National Center for Laboratory Animal Breeding, the Center for Genetic Engineering and Biotechnology, and Hermanos Ameijeiras Hospital (Havana, Cuba). The subjects provided written informed consent for the investigational use of their surgically excised material. These included healthy tissue (dermis and epidermis) serving for control groups derived from healthy female donors undergoing abdominal and facial cosmetic surgery. Malignant samples used in the study consisted of: (1) - Three triple-negative mammary invasive ductal carcinomas (IDCs), which resulted in a high histological grade and intense mitotic index with lymphatic/vascular permeation and confirmed the invasion of sentinel lymph nodes. Donors were white females of ages 34–46 years. (2) - A histologically well-differentiated pancreatic ductal adenocarcinoma tissue was obtained from a 56-year-old white male. (3) - A metastatic melanoma tissue in a cervical lymphatic ganglion was obtained from a 32-year-old black female patient. All the samples were collected during the surgical intervention, washed with ice-cold sterile normal saline to remove fibrin and debris, and cryopreserved in liquid nitrogen until processing for CFF preparation. Tumor sample fragments were fixed in 10% buffered formalin and paraffin-processed for histological analysis, as per protocol. The oncologic samples were ultimately processed and used for the experiments, having received pathology reports of malignancy.

### Preparation of cell-free filtrates

The collected tissue was allowed to thaw and weighed, and approximately 100 mg of wet tissue was placed in a 2-mL vial containing 1 mL of normal saline, which was homogenized using a TissueLyser II for 3 min at 30 revolutions per second. The samples were then centrifuged at 10,000 rpm for 10 min at 4°C, sterilized by filtration using 0.2-μm nitrocellulose filters (Sartorius Lab Instruments), aliquoted into sterile Eppendorf vials, and stored at −70°C. Given the histological similitude of the IDC samples, the three tumor samples were pooled to ensure larger material availability. For the three study protocols described here, protein concentration was used as the arbitrary unit of measurement to prepare and administer the inoculums.

### CFF biochemical characterization

All the biochemical parameters were determined by spectrophotometric methods using commercial kits. Pro-inflammatory markers included C-reactive protein (CRP) [C-Reactive Protein (PTX1) Human ELISA Kit, Abcam, Cambridge, United Kingdom], interleukin (IL)-1β (IL-1 beta Human ELISA Kit, Abcam, Mass, United States), IL-6 (IL-6 Human ELISA Kit, Abcam), and tumor necrosis factor α (TNFα) (Human TNF alpha ELISA Kit, Abcam). Oxidative stress markers included malondialdehyde (MDA) [Lipid Peroxidation (MDA) Assay Kit, Abcam] and H_2_O_2_ (Hydrogen Peroxide Assay Kit, Abcam). Additionally, Sirtuin-1 (SIRT1) levels were determined using the ELISA Kit for SIRT1 (Cloud-Clone Corp., Houston, Texas, United States). In all cases, the manufacturer’s instructions were followed.

### Animals

The protocols involving the use of rodents described here were conducted with male BALB/c-Foxn1nu/Cenp mice (body weight 19–22 g and age 12 to 14 weeks) obtained from the National Center for Laboratory Animal Breeding (CENPALAB, Havana, Cuba) and maintained in ventilated racks (Tecniplast, Varese, Italy) in certified rooms for nude mice. Autoclaved food EAO 1004 (CENPALAB, Havana, Cuba) and water were provided *ad libitum*. Room temperature (20°C–23°C), humidity (65% ± 10%), and the photoperiod cycles (12 h per day) were automatically controlled. The animals were observed twice a day by an experienced staff for monitoring their health status. Body weight was registered a day before study commencement, on a weekly basis, and before the autopsy.

Wild-type adult zebrafish (*Danio rerio*) were maintained in a recirculating aquatic system facility at the Drug Research and Development Center (CIDEM) under controlled environmental conditions, including a 14-h light/10-h dark cycle, water temperature of 28°C, and pH 7.2–7.5. The fish were fed commercial zebrafish food twice daily, and the system water quality parameters were regularly monitored to ensure optimal breeding conditions and embryo development according to the internal operational procedures.

All the animal procedures were performed according to local and International Guiding Principles for Biomedical Research. All animal studies were conducted under a protocol approved by the Institutional Animal Care and Use Committees of the National Center for Laboratory Animal Breeding (CENPALAB), the Center for Genetic Engineering and Biotechnology, and CIDEM.

## Experimental protocols

Three study protocols corresponding to each tumor histotype CFF were followed. The study examined the effects of 1) breast tumors, 2) pancreatic ductal adenocarcinoma, and 3) metastatic melanoma. For each protocol, 24 BALB/c-Foxn1nu/Cenp mice were randomly distributed into two experimental arms (N = 8/arm) receiving: (1) CFF derived from malignant tissue samples, (2) control CFFs derived from healthy donor skin. The mice in protocol I received 100 µg of protein (as an arbitrary unit) of normal skin tissue or breast malignant samples, whereas for protocols II and III, the protein concentration was reduced to 50 µg—all in a volume of 250 µL of normal saline once a day from Monday through Saturday for 12 weeks as per the initial schedule. However, the administration period was reduced to 6 or 8 weeks due to the progressive morbidity of the mice receiving the CFFs prepared with metastatic melanoma and pancreatic adenocarcinoma tissues. We assumed this administration time window for the three mice protocols as it proved to induce malignant changes in a previous study (Berlanga-Acosta et al., 2022c). Subcutaneous and intraperitoneal routes were alternatively used on a weekly basis to prevent local injury due to repeated trauma.

A fourth experimental protocol was mostly addressed to examine whether the incubation of cells with malignant tissue-derived homogenates elicits the expression of tumor markers. For this purpose, sets of zebrafish embryos were incubated with breast ductal carcinoma tissue, and healthy donor skin homogenates were used as the primary control. Other controls included incubation in system water alone and normal saline as the homogenate vehicle. Each experiment was performed in triplicate, and a minimum of 10 embryos were used per treatment group. Upon the sixth hour post-fertilization (hpf), the embryos were exposed to 100 μg of protein/mL until hour 66 of embryonic development. This concentration dose proved to be the maximal tolerated dose with no lethal morphogenetic anomalies, according to a dose–response pilot experiment. In addition to the immunocytochemical expression of tumor markers, we also recorded the hatching rate, morphological anomalies, and heartbeat frequency. During the exposure period, the embryos were regularly inspected by qualified specialists (YNF and SHP) in search of developmental changes and heartbeat counting, as previously described (Holden et al., 2013; Gaur et al., 2018).

### Mice autopsy, tissue processing, and immunohistochemistry

The animals were euthanized under terminal anesthesia at the end of the administration period. Alternatively, mice that evolved to an extreme clinical condition during the administration period were sacrificed to ensure a proper autopsy and organ collection (as described below). The autopsy study was conducted following an internal protocol based on described techniques (Scudamore et al., 2014). Gross noticeable changes in organs and tissues were recorded, and fragments were collected for histopathological analysis. Representative fragments from apparently healthy organs were also harvested. Samples were fixed in 10% buffered formalin and paraffin-embedded, and serial 5-μm sections were stained using H&E. Images were captured using a BX53 Olympus microscope coupled to a digital camera and central command unit (Olympus Dp-21). Histological examinations were blindly performed by three MD pathologists experienced in experimental pathology (EAH, DDC, and LLM). The histopathological findings of premalignant and malignant lesions were collectively discussed and ultimately diagnosed in accordance to the current recommendations (Nikitin et al., 2004; NTP, 2023). Paraffin sections of representative tissue lesions in mice receiving the tumor material and the corresponding specimens from control counterparts were mounted on poly-l-lysine-coated slides (Dako, California, United States) in order to reduce inter-tissue/experimental variations during immunohistochemistry studies. The slides were dewaxed and rehydrated through graded washes with ethanol. The rehydrated slides were exposed to a high-pH antigen retrieval solution (Dako, United States) for 20 min at 90°C. Following equilibration at room temperature, the slides were washed in PBS and blocked with endogenous peroxidase. Unspecific binding blocking solution was used for 20 min, and the sections were incubated for 40 min with antibodies directed to TTF-1 (1/100; Santa Cruz Biotechnology SC-53136), c-Myc (1/100; Abcam, ab32072), proliferating cell nuclear antigen (PCNA) (1/250; Cell Signaling Technology (PC10) Mouse mAb #2586), estrogen receptor (1/250; Santa Cruz Biotechnology (D-12) SC-8005), progesterone receptor (1/250; Santa Cruz Biotechnology, FKBP51 SC-271547), HER-2 (1/200; Santa Cruz Biotechnology, ErbB2/HER2 (A-2) SC-393712), telomerase reverse transcriptase (TERT) (1/150; Abcam, ab216625), survivin (1.200; Abcam, ab469), CD56 (1/200; Invitrogen, Cat #PA5-78402), CD45 antigen (leukocyte common antigen, 1/100; Abcam, ab10558), and cytokeratin 7 (CK7) (1/250; Abcam, ab181598). Antigens were retrieved according to each antibody manufacturer’s instructions and protocol. The immunolabeling reaction was developed as described for the Mouse- and Rabbit-Specific HRP/DAB (ABC) Detection IHC kit (Abcam, ab64264). Non-specific tissue labeling internal controls included the omission/replacement of the primary antibody by the background-reducing antibody diluent and normal rabbit serum (Boster Biological Technology, Pleasanton, CA, United States, catalog # AR1010).

### Zebrafish embryo immunocytochemistry

After 60 h of incubation with the pathologic and healthy tissue-derived homogenates and the other controls, the embryos were processed for immunocytochemistry, as previously described (Guille, 2008). In brief, embryos were rinsed with PBS and fixed in 4% paraformaldehyde at 4°C for 1 h and post-fixed in acetone at −20°C on poly-l-lysine-coated slides. Before incubation with the primary antibody, the embryos were soaked in a background-reducing protein block (Abcam 156024) for 15 min at room temperature. Subsequently, the embryos were incubated with anti-c-Myc (1/250) and anti-HER-2 (1/500) antibodies for 20 min at room temperature. After three washes with PBS, the slides were incubated with a biotinylated secondary linker and washed with horseradish peroxidase (HRP)-labeled streptavidin solution, as previously described (Abcam, 2024). Primary antibody binding is identified by the reddish-brown stain due to the HRP reaction with diaminobenzidine. The reaction development was stopped by PBS washes, and the coverslips were mounted with ClearMount™ Mounting Solution (Invitrogen). No contrast was used. Immunolabeled cells were identified through anatomic illustrative descriptions of zebrafish embryos (von Hellfeld et al., 2020).

### Statistical processing

Statistical analyses were performed using GraphPad Prism 10.1.0 software. Normal distribution was analyzed using D’Agostino–Pearson and Shapiro–Wilk normality tests. Variance homogeneity was evaluated using Brown–Forsythe and Bartlett’s tests. The mice groups were compared during the study using two-way ANOVA, followed by Tukey’s multiple comparisons test. Body weight changes were processed, and inter-groups were compared through the mixed-effects model (REML) method and Dunnett’s multiple comparisons test. Survival data were analyzed using the log-rank Mantel–Cox test. *p*-values < 0.05 indicated statistically significant differences.

## Results

### Cell-free filtrate descriptive biochemical characterization

As described, surgical pieces of healthy donor skin and fragments of three different human malignant tumors were used to prepare the CFFs. The elemental biochemical description shown in Table 1 indicates that cellular lipids undergoing a peroxidation process are similar among the healthy skin and the tumor samples, as reflected by the MDA levels. However, metastatic melanoma generates superoxide anion and hydroxyl radicals, as judged by the H_2_O_2_ levels, which exceeded the values determined for the healthy control skin and other malignant samples by between 4 and 6-fold. Tumor-derived samples seemed to exhibit a particular inflammatory signature. CRP appeared particularly elevated in pancreatic adenocarcinoma cells, exceeding the concentration detected in metastatic melanoma and healthy control skin by over 24-fold. Melanoma-derived CFFs, however, showed the highest level of IL-6, followed by pancreatic adenocarcinoma cells. In line with this, melanoma also exhibited the largest concentration of IL-1β, TNFα, and Sirtuin-1, which was followed by the concentration determined in mammary carcinoma homogenates. Of note, healthy donor skin tissue homogenates showed the lowest values of IL-1β, while IL-6, TNFα, and Sirtuin-1 were not detected (Table 1).

**TABLE 1:** Biochemical characterization of the cell-free filtrates (CFFs).

Sample	Malondialdehyde (MDA) (μM)	H_2_O_2_ (pmol/μL)	C-reactive protein (CRP) (ng/mL)	Interleukin 6 (IL-6) (pg/mL)	interleukin-1β (IL-1β) (pg/mL)	Tumor necrosis factor alpha (TNFα) (pg/mL)	Sirtuin 1 (ng/mL)
Control healthy donor skin	3.47	17.39	21.95	ND	25.80	ND	ND
Mammary ductal carcinoma	3.89	25.88	152.39	30.15	116.18	72.89	17.21
Pancreatic ductal adenocarcinoma	3.05	21.66	529.75	151.39	99.70	27.25	5.30
Metastatic melanoma	4.20	108.43	18.35	262.40	807.24	127.18	23.01

ND, not detectable.

### Protocol 1: breast tumor CFF administration

**FIGURE 1:**
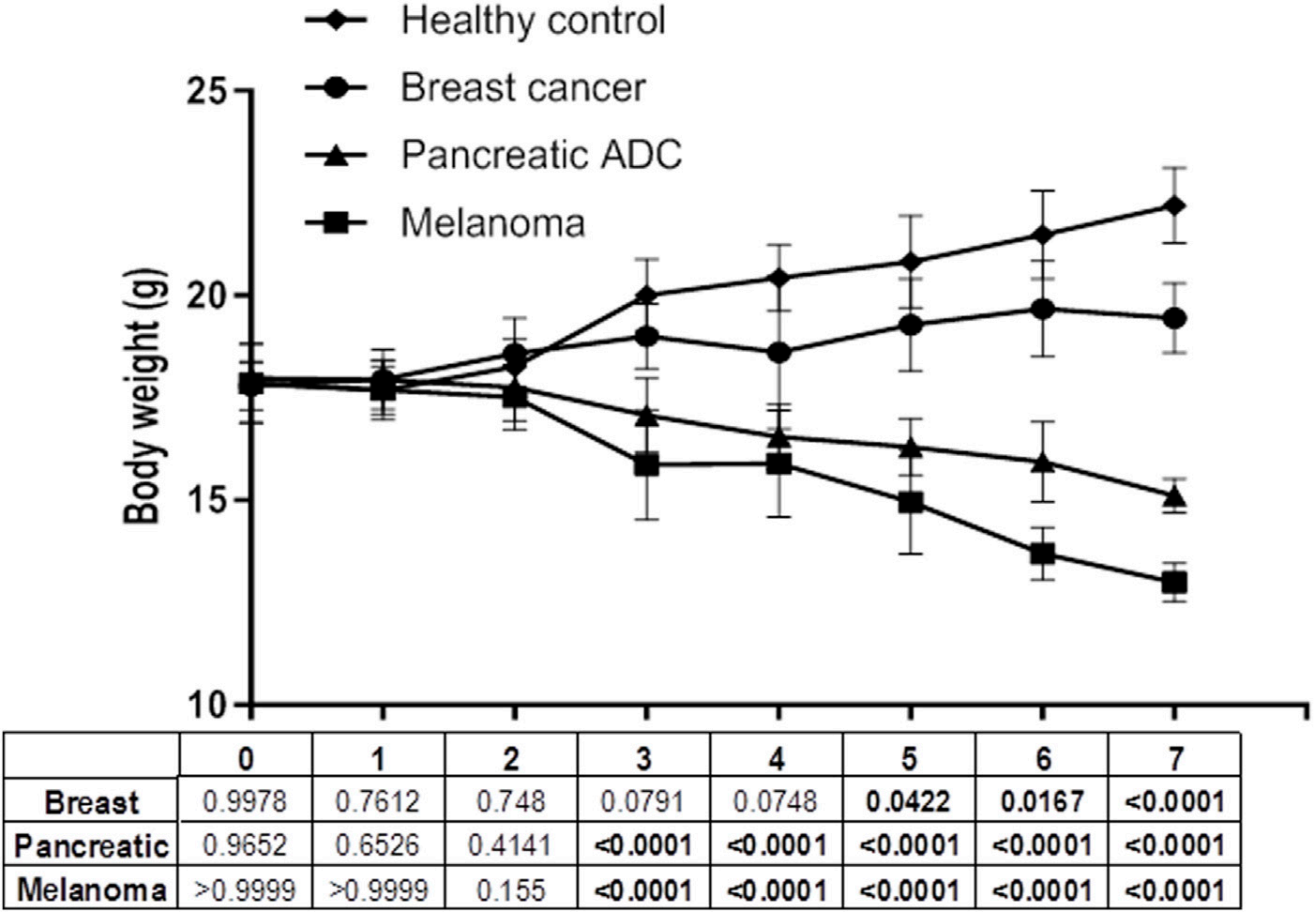
Body weight evolution during the administration period. Body weight changes were processed and inter-groups compared through the mixed-effects model (REML) method and Dunnett’s multiple comparisons test. Statistical significance was established for *p* < 0.05.

The administration of mammary ductal carcinoma CFFs was not associated with behavioral changes, clinical deterioration, or severe/acute somatic emaciation. As shown in Figure 1, the effect of the mammary carcinoma homogenate significantly impacted the animal body weight from the fifth week onward compared to the recipients of the healthy donor homogenates. This mammary cancer CFF did not affect animal survival during the effective administration period of 8 weeks.

**FIGURE 2:**
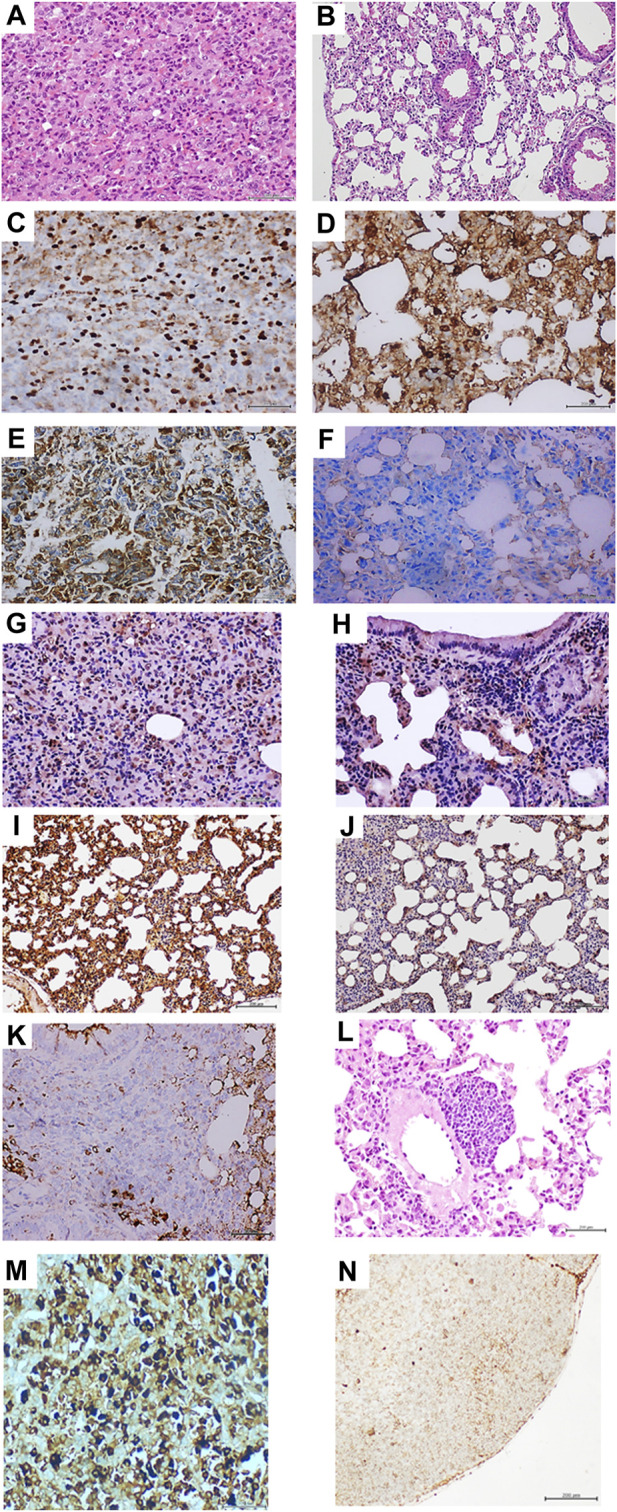
Histopathological and immunohistochemical characterization of lung tumors in mammary carcinoma-treated mice. **(A)** Histological image representative of the solid growth pattern of lung adenocarcinoma in animals treated with the homogenate of triple-negative breast tumor. The intense cellular proliferation erased the alveolar lumen, which is in sharp contrast with the normal aspect of lung parenchyma in animals from the control group, as shown in **(B)** hematoxylin/eosin (H/E) ×40. **(C)** An intense and broadly distributed nuclear immunohistochemical expression of thyroid transcription factor 1 (TTF-1) was observed in the lung adenocarcinomas of the mammary tumor-treated mouse group. **(D)** shows that few stained TTF-1-positive nuclei are counted in the alveolar walls of the control group ×40. Tumor-treated mouse lung samples were positive for HER-2 expression, basically in the cytoplasm and cell membrane in **(E)**, whereas this marker was not expressed by lung cells of mice from the control group **(F)**, ×40. Large and intense nuclear immunolabeling was found for the cell proliferation marker proliferating cell nuclear antigen (PCNA) by the tumor cells as shown in **(G)**. Lung cells from the control group exhibited a far more restricted expression in alveolar walls **(H)**, ×20. As shown in **(I)**, broad and intense oncogene c-Myc expression was observed in normal alveolar walls adjacent to the adenocarcinoma nodules. Conversely, lungs from control mice **(J)** showed faint and marginal immunolabeling × 20. **(K)** demonstrates that within the periarteriolar and peribronchiolar rosette-like conglomerates, some atypical round, small, blue cells were positive for CD56 signals, indicating a neuroendocrine line, × 40. The presence of a nodule made up by round, blue cells suggestive of a small-cell carcinoma is shown **(L)** attached to the adventitia of a large vessel, H/E × 20. Mediastinal lymphatic nodes of mice treated with the breast tumor homogenate exhibited cells immunoreactive to cytokeratin 7, HER-2, and hormone receptors. Here, an intense and specific nuclear signal for the progesterone receptor is shown in **(M)**. This reaction contrasts with the negligible immunolabeling shown by the node lymphatic cells shown in **(N)** from control mice receiving normal skin homogenates. Furthermore, an appreciable difference in nuclear size between both samples was observed. Sections were 5 µm. Magnification ×40 for both. Scale bar: 200 µm.

The administration of breast carcinoma homogenates generated premalignant and malignant changes in lung tissue. Microscopic examination of the lungs in all these mice (N = 8) exhibited extensive areas of parenchymal condensation, in which atypical adenomatous hyperplasia (AAH) predominated at the expenses of type 2 pneumocytes with clear, enlarged, and atypical nuclei (not shown). In these areas of parenchymal condensation and alveolar luminal encroachment, large subpleural nodules diagnosed as adenocarcinoma of both solid and lepidic growth were distinguished (Figure 2A). Mice treated with the healthy skin homogenate showed morphologically normal pulmonary parenchyma (Figure 2B). The immunohistochemical characterization of these nodules confirmed their authentic malignant nature. Tumor cells exhibited a strong nuclear signal for TTF-1 (Figure 2C), which contrasted with the restricted expression of the control specimens (Figure 2D). These cells also exhibited remarkable cytoplasmic HER-2 expression (Figure 2E) compared to controls (Figure 2F).

Broad nuclear expression of PCNA was observed in the tumor-bearing mice (Figure 2G) compared to the expression in the lung normal parenchyma of healthy skin-treated mice (Figure 2H). Nuclear and cytoplasmic c-*Myc* overexpression that extended to areas of microscopically normal alveolar walls was found in the lungs of breast-tumor inoculated mice (Figure 2I). Control mice alveolar walls, however, exhibited only marginal immunolabeling (Figure 2J). Meaningful nuclear TERT expression was observed in these tumors, concurrent with the overexpression of nuclear survivin, nuclear estrogen, and progesterone receptors as part of the tumor phenotypes (not shown). Within these tumors, conglomerates of cells appeared organized, forming rosette-like structures that encircled the arterioles and bronchioles. The rosette-like aggregates were heterogeneous and contained large, atypical, irregular, epithelial, CK7-positive cells (not shown) and small, round, blue cells that often appeared with large-sized vesiculous nuclei with lax chromatin and central conspicuous nucleoli. Immunohistochemistry demonstrated that some of these cells were negative for CD45 (not shown), whereas some cells were found positive for CD56, suggesting its neuroendocrine origin (Figure 2K). These round blue cells were often visualized forming subpleural, periarteriolar, and peribronchial nodules with notorious nuclear molding, presumptively identified as small-cell lung carcinoma (Figure 2L).

Finally, an interesting immunohistochemical finding was the presence of HER-2, CK7, estrogen, and progesterone receptor-positive cells in the subcapsular sinus of thoracic lymphoid nodes. Intense nuclear labeling for both hormone receptors was observed in these cells. Figure 2M shows the intense expression of the progesterone receptor; lymphatic node samples from the mice control group were negative for HER-2 and CK7. Faint staining for progesterone (Figure 2N) and estrogen receptors appeared circumscribed to the cell membrane.

### Protocol 2: pancreatic ductal adenocarcinoma CFF administration

After 6 injections of the tumor CFF, animals began to show asthenia, a tendency of isolation, hunched posture, and frail, dry, and flaky skin. Although the dose was de-escalated to 50 µg in the second week in order to prevent animal decay, the aspect of “ill condition” continued with evident emaciation. Severe and progressive cachexia debuted in the third week. A significant body weight loss was observed from that point onward compared to healthy control recipients (Figure 1). Four animals with ostensible clinical deterioration were untimely euthanized and autopsied, whereas the other four mice, although in a cachectic state, completed 8 weeks of administration. Healthy donor skin-treated mice remained active and healthy during the administration period.

**FIGURE 3:**
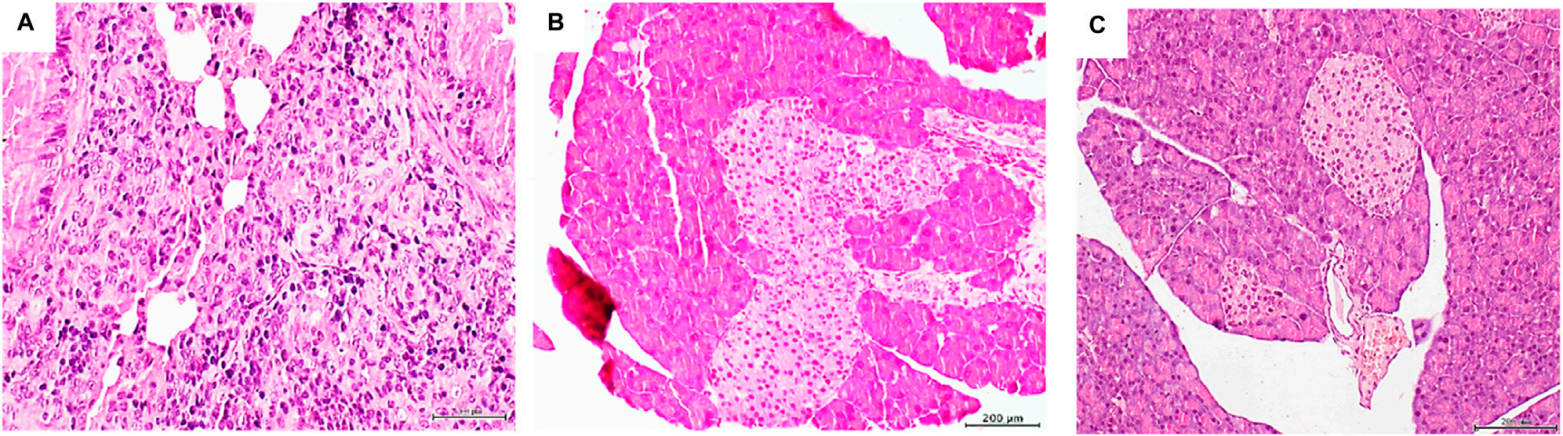
Histopathological changes detected in mice treated with the pancreatic adenocarcinoma homogenate. **(A)** Histological panoramic image representative of the poorly differentiated, solid adenocarcinomas found in mice treated with the pancreatic adenocarcinoma homogenate. Matrix disorganization, cellular overcrowding, nuclear pleomorphism, and atypia were observed. H/E ×20. The pancreatic tumor homogenate also impacted the mouse pancreas, shown by a remarkable Langerhans islet hypertrophy, as shown in **(B)**. Pancreas of those mice treated with the healthy skin homogenate exhibited a normal aspect on the islets and the pancreas in general **(C)**. Sections were 5 µm. H/E staining. Magnification × 10. Scale bar: 200 µm.

The histopathologic examination of mice organs exposed to pancreatic tumor CFFs revealed that, again, the lungs appeared to be the major target structure with pre- and malignant changes. Two major findings were obtained: multiple foci of solid, poorly differentiated adenocarcinoma across the lung parenchyma tissue (Figure 3A) and marked hyperplasia of pancreatic Langerhans islets (Figure 3B). Alveolar/bronchiolar hyperplasia and atypical adenomatous hyperplasia (AAH) were the only findings attributable to the pancreatic tumor CFF in mice that were prematurely autopsied. Nevertheless, immunohistochemistry revealed a strong nuclear and cytoplasmic TTF-1 expression in the bronchoalveolar cells within these foci of AAH (not shown). None of the mice treated with the healthy donor skin homogenates exhibited microscopic pathologic changes, including the normal aspect of Langerhans islets (Figure 3C).

### Protocol 3: metastatic melanoma CFF administration

Closely resembling the effect of the pancreatic tumor homogenate, the administration of 50 µg of melanoma-derived protein also led to a cachectic process and clinical involution from the third week of treatment onward (Figure 1). Consequently, the mice were untimely and gradually autopsied on days 34, 36, 43, 45, 49, and 55 due to their morbid clinical aspect. On day 61, the only two remaining mice were autopsied, and the administration period was concluded for this protocol. The mice treated with the healthy donor skin remained normal and active during the 2-month administration period. Two mice of this group were found dead on days 57 and 60 with evidence of autolysis.

**FIGURE 4:**
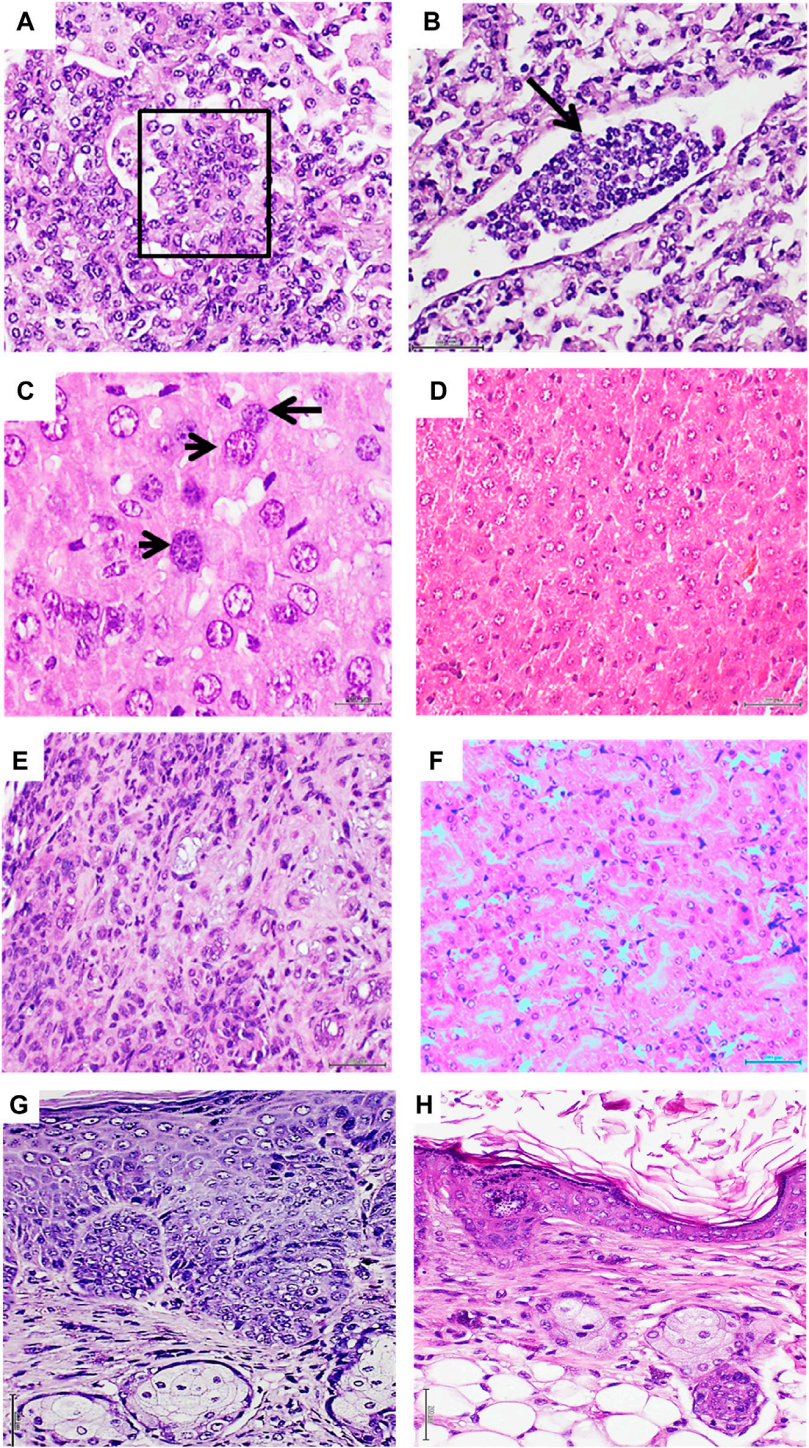
Histopathological changes detected in mice treated with metastatic melanoma homogenates. **(A)**: Histological image representative of a solid adenocarcinoma with pseudopapillary growth patterns in a mouse treated with the metastatic melanoma homogenate. The papillae within the box show the overlapping of atypical nuclei. Correspondingly, a conglomerate of heterogeneous cells with scant cytoplasm and occupying the lumen of a lymphatic vessel is shown in **(B)** (black arrow). The cells are morphologically similar to those observed in tumor nodules and papillae, suggesting the migration/circulation of tumor-derived cells. Both H/E ×20. Melanoma CFFs induced enlargement of hepatic cells and their nuclei. As shown in **(C)**, enlarged, atypical, and variegated nuclei with a coarse chromatin were found across the liver parenchyma (black arrow). A normal aspect of the liver parenchyma derived from normal skin-treated mice is shown in **(D)**. Both H/E ×40. Infiltration of neoplastic cells in kidneys from melanoma-treated mice **(E)** involved the interstitium, peritubular capillaries, and tubular lumen. An interstitial inflammatory infiltrate with a desmoplastic reaction was observed. This image of matrix disorganization and cellular pleomorphism is fully divergent with the aspect of normal kidney parenchyma from the control mice, as shown in **(F)**. Both H/E ×20. **(G)** An aberrant and irregular proliferation of epidermal cells with prominent nuclear atypia, overcrowding, and mitotic figures was induced by the melanoma homogenate. A difference in the epidermal layer cellularity and organization corresponding to control group mice was observed, as shown in **(H)**. Both H/E ×20. Sections were 5 µm; scale bar: 200 µm.

Melanoma-derived CFF administration, in addition to exerting the described cytotoxic effect, induced a broad spectrum of pre-malignant and malignant proliferative changes that involved the lungs, liver, kidneys, and skin. Bronchoalveolar proliferative changes extended from AAH to intra-alveolar pseudopapillary adenocarcinomas, which were made up of round blue cells with atypical and overlapping irregular nuclei, exhibiting hyperchromasia and clumped chromatin. Another cell population observed in this conglomerate showed large vesiculous nuclei with a coarse dispersed chromatin and prominent nucleoli (Figure 4A). In line with this pathologic proliferation in the lungs is the identification of circulating tumor cells as an embolus within a lymphatic vessel lumen, integrated by a cluster of cells with the same cytological traits as described above (Figure 4B). In relation to the liver, two major alterations were identified (Bordonaro, 2019): the presence of nodules or aggregates of cells of putative leukocytic origin with small, irregular, and atypical nuclei scattered across the liver parenchyma (not shown) (NCI, 2021) and the presence of voluminous hepatocytes with large, atypical, and variegated nuclei containing a coarsely granular or dispersed chromatin (Figure 4C). Normal liver histology was observed in the group of control mice (Figure 4D). In the kidney parenchyma, infiltration of neoplastic cells was found in the interstitium, peritubular capillaries, and the lumen of the tubules, demonstrating cellular atypia, irregular nuclei, pleomorphism, and prominent nucleoli. An interstitial inflammatory infiltrate of lymphocytes and polymorphonuclear neutrophils with a desmoplastic reaction was also observed (Figure 4E). On the contrary, animals from the control group showed no pathological changes in kidney parenchyma (Figure 4F). Skin of melanoma-treated mice also revealed an abnormal and irregular epidermal proliferative disorder, which included the epidermis and epithelial dermal ridges. Epidermal cells showed notable nuclear overlapping, the presence of irregular, sharply angulated, and hyperchromatic nuclei, mitotic figures, and prominent nuclear atypia (Figure 4G). The skin of the control mice showed no histopathological alterations (Figure 4H).

### Protocol 4: zebrafish embryos express tumor markers

**FIGURE 5:**
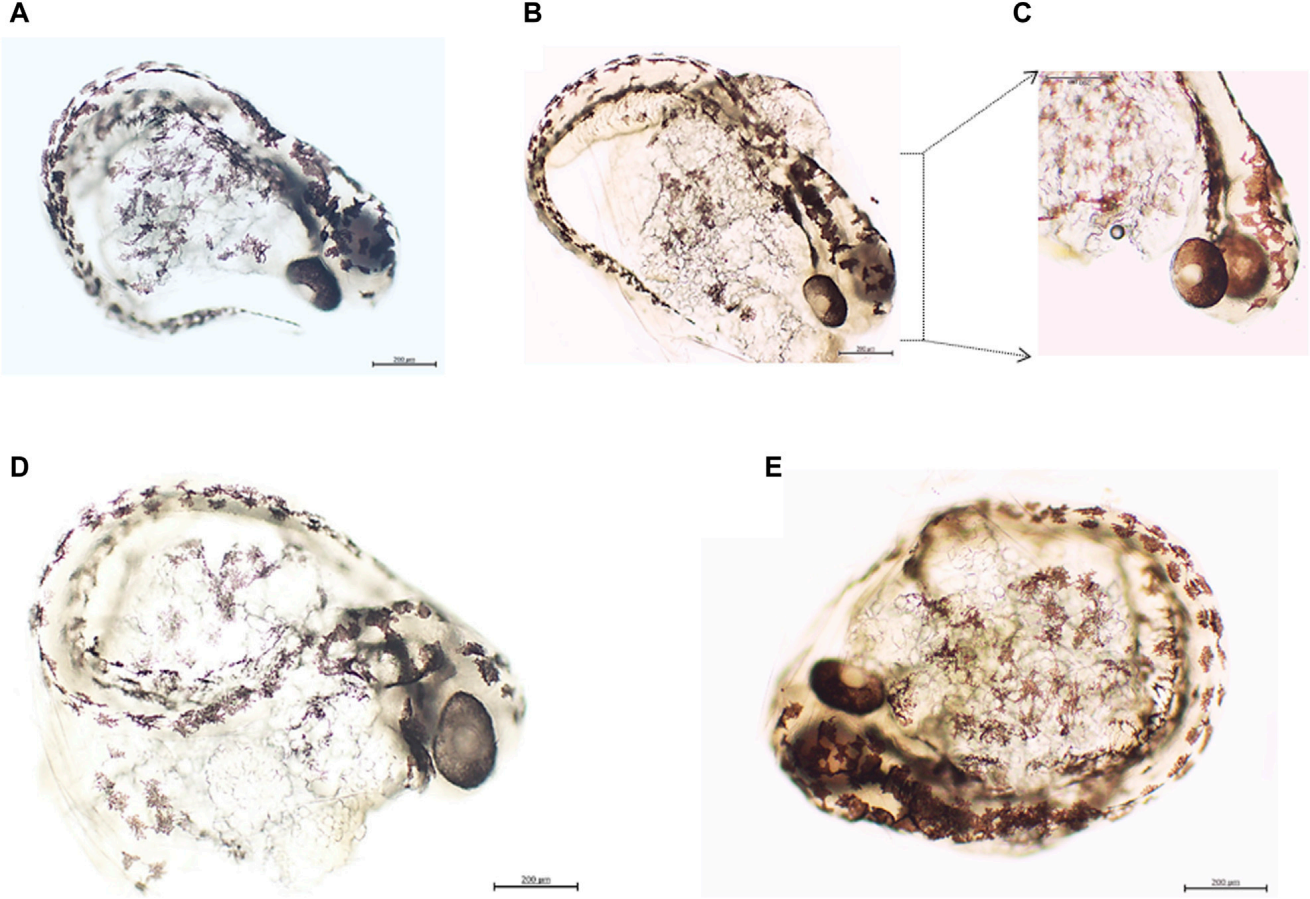
Panels 5A–5E correspond to the fourth experimental protocol. Expression of tumor markers by zebrafish embryos after incubation with malignant tissue-derived homogenates. **(A)** Embryo exposed to the healthy donor skin homogenate and incubated with an anti-c-*Myc* antibody. A light background is observed along the line of melanocytes upon the notochord. Magnification ×10. **(B)** Embryos exposed to breast ductal carcinoma homogenates. c-*Myc* expression by the same melanocytes population and other cells of the cephalic pole was observed. Magnification ×10. **(C)** c-*Myc* expression by the eye cells and cells over the area of the rhombencephalon, cerebellum, and tegmentum. Magnification ×20. **(D)** Embryos treated with healthy donor-skin homogenates and incubated with an anti-HER-2 antibody. Again, melanocytes and cephalic pole cells exhibit a faint expression, which is in contrast with the stark expression detected in sample **(E)** corresponding to embryos exposed to breast ductal carcinoma homogenates. Melanocytes and notochord cells, alike cells on the cephalic pole over areas of the rhombencephalon, cerebellum, epiphysis, and optic tectum, express HER-2. All the embryos were observed approximately 66–68 h post-fertilization. Magnification ×10. Scale bar: 200 µm.

The incubation of zebrafish embryos with 100 μg/mL of protein from breast ductal carcinoma homogenates for approximately 60 h proved to elicit the expression of c-M*yc* and HER-2 in stark contrast to embryos exposed to healthy donor skin (Figure 5). A constellation of embryo cells was found to express both tumor markers. These included melanocytes, notochord, cephalic, and eye cells. Of note, incubation with malignant tissue homogenates acutely altered the embryo homeostasis. The number of heartbeats/minute was significantly reduced at the 24-h time point and during the two subsequent evaluation points. Other morphological non-lethal anomalies, such as hypopigmentation and spine curvature, were observed at 48 and 66 h of evaluation. Exposure of embryos to healthy donor-derived CFFs (control of human material) did not induce functional or morphological changes in the embryos, similar to the two other controls included (Table 2).

**TABLE 2:** Functional and morphological evaluation of embryos incubated with human pathologic and healthy donor-derived homogenates.

Time*	Variables	Aquarium water control	Saline control	Healthy donor skin	Breast ductal carcinoma
24 h	Heart rate (min^-1^)	92 ± 6 **(a)**	87 ± 7 **(a)**	87 ± 6 **(a)**	59 ± 6 **(b)**
	Hatched embryos	0/10	0/10	0/10	0/10
	Spine curvature	0/10	0/10	0/10	0/10
	Hypopigmentation	0/10	0/10	0/10	5/10
	Healthy embryos	10/10	10/10	10/10	10/10
48 h	Heart rate (min^-1^)	157 ± 7 **(a)**	159 ± 8 **(a)**	157 ± 6 **(a)**	131 ± 5 **(b)**
	Hatched embryos	0/10	0/10	0/10	5/10
	Spine curvature	0/10	0/10	0/10	5/10
	Hypopigmentation	0/10	0/10	0/10	4/10
	Healthy embryos	10/10	10/10	10/10	5/10
66 h	Heart rate (min^-1^)	157 ± 3 **(a)**	160 ± 4 **(a)**	159 ± 5 **(a)**	132 ± 6 **(b)**
	Hatched embryos	8/10	8/10	9/10	9/10
	Spine curvature	0/10	0/10	0/10	6/10
	Hypopigmentation	0/10	0/10	0/10	0/10
	Healthy embryos	10/10	10/10	10/10	4/10

*Time: hours of evaluation after incubation with pathologic or normal donor-derived homogenates. Statistical analysis of the heart rate was performed using two-way ANOVA followed by Tukey’s multiple comparisons test. Different letters mean significant statistical differences among treatments in the same time point (*p* < 0.01).

## Discussion

To experimentally examine the hypothetical existence of soluble and transferable tumor tissue-derived drivers, crude homogenates from fresh human tumors were inoculated in nude mice; triple-negative invasive ductal mammary carcinoma, pancreatic ductal adenocarcinoma, and a metastatic melanoma are three varieties of poor prognosis cancers with limited treatment options, ability to develop resistance to therapies, and dismal 5-year survival rates (Arnold et al., 2022; Schroder et al., 2022; Siegel et al., 2022; Yeo et al., 2022). The hypothesis was also examined through the incubation, for a period of hours, of pre-hatched zebrafish embryos with the breast cancer homogenates and concurrent controls.

Irrespective to its limitations, this study confirms, extends, and, consequently, supports our hypothesis that human malignant tissue-derived homogenates disrupt the proliferative and differentiation programs of normal host cells, leading to the onset of premalignant and malignant changes. A previous study by our group addressing the effects of 3 months of administration of an invasive mammary carcinoma and a pleomorphic sarcoma-derived CFF proved that they act as a genuine carcinogen in nude mice, rendering epithelial and mesenchymal tumors that exhibited irreversible, metastasizing, and autonomous progression from the sixth week onward (Berlanga-Acosta et al., 2022c). The findings obtained in the present study while administering the breast carcinoma-derived CFF to mice faithfully reproduce and converge with these previous pathologic descriptions (Berlanga-Acosta et al., 2022c). We deem this a meaningful event, given that the multidimensional complexity of cancer has historically caused variability, heterogeneity, and lack of reproducibility in basic research experiments (Alekseenko et al., 2023).

The experimental methodology used in this opportunity was as previously described (Berlanga-Acosta et al., 2022c); tumor-derived CFFs were prepared using a sterile physiologic saline solution with no purification processes or any other type of chemical manipulation. These CFFs are homogenates that represent a pure extract of the pathologic tissues, are rich in content material, and are a vehicle of donor cell soluble signatures that have previously been proven to recapitulate in healthy recipient rodents the histopathologic hallmarks of diabetic angiopathy and neuropathy and non-diabetic-related arteriosclerosis as models of non-communicable chronic diseases (Berlanga-Acosta et al., 2021; Berlanga-Acosta et al., 2022b). Of note, we described that despite the mechanical processing of tissue disruption to elaborate the CFFs, molecules of DNA and RNA from the tumors are detected in the homogenates, and RNA may be successfully reverse-transcribed and its product amplified. This fact supports our hypothesis of a possible uptake by the host normal cells of some sort of free or exosome-encapsulated tumor-derived genetic or epigenetic carcinogenesis primer, i.e., a horizontal transference of genetic material (Berlanga-Acosta et al., 2022c).

Given that none of these alterations were identified in any of the control animals, it suggests that there was no spontaneous tumorigenesis during the experimental period, and that the neoplastic traits observed do not represent a form of tissue reactive response to the human xenogeneic material. This later contention is supported by the following observations: (1) pathologic consequences of tumor-homogenates in animals involved a microscopically typical disorder of cellular proliferation and differentiation. (2) The transformed tissues adopted an abnormal immunohistochemical profile, typical of legitimate malignancies. (3) The absence of a reactive, immunoinflammatory response was associated with the human material inoculation or the tumor itself.

The simple and descriptive biochemical characterization of the three tumors and the healthy skin used as control indicated that tumors, particularly metastatic melanoma, exhibit elevated peroxidative and pro-inflammatory profiles. Melanoma-derived CFFs presented the highest concentrations of H_2_O_2_ and IL-6, IL-1, TNFα, and Sirtuin 1. These are all well-renown tumor biomarkers with a definitive role in carcinogenesis promotion, malignant progression, cancer invasiveness, chemotherapy resistance, and metastasis, and, in general, correlate with a worse prognosis (Berraondo et al., 2019; Costa-Machado and Fernandez-Marcos, 2019; Yang et al., 2020; Propper and Balkwill, 2022; Wen et al., 2022). Conversely, most of these biomarkers were not detected in control healthy skin-derived homogenates. According to the clinical evolution of the animals, it is tenable to suggest that the pro-oxidative profile and the inflammatory signature of the tumors exerted toxic imprinting in the recipient mice, which may explain the differences observed in the clinical evolution and the pathologic spectrum in each experimental protocol. This hypothesis is supported by the clinical reaction and the progressive mortality registered in mice receiving the melanoma and by those treated with the pancreatic adenocarcinoma, even when the initial dose was de-escalated to prevent the collapse of the experiment. Furthermore, animals treated with melanoma CFFs also showed a broader profile of proliferative changes encompassing the lungs, liver, kidneys, and skin, whereas the impact of pancreatic and breast tumors appeared circumscribed to the lungs. Aside from the clinical toxicity and the cachectic syndrome imposed by the melanoma and the pancreatic adenocarcinoma compared to the effect of breast carcinoma, a common pathogenic outcome was the induction of *bona fide* lung adenocarcinomas, frequently classified as poorly differentiated. We had previously observed this type of lung tissue tropism in mice treated with breast carcinoma and pleomorphic anaplastic sarcoma CFFs (Berlanga-Acosta et al., 2022c). The signalers and mechanisms behind this particular tissue tropism for lungs remain intriguing to us.

It is also difficult to explain why breast carcinoma-treated mice having large areas of lung parenchymal condensation by tumoral growth remained clinically normal and not emaciated. A simple interpretation of this observation is that these were “indolent-like tumors,” and that it is one thing to host a malignant tumor and another thing is to be sick with cancer. An interesting and unusual finding observed in three mice treated with breast carcinoma is the coexistence of two histologically different tumors within the same lung. On one side, solid and lepidic patterns of adenocarcinomas overexpressing a set of well-validated malignancy immunohistochemical markers were observed (Yuan and Xu, 2019; Wang et al., 2020; Gloriane et al., 2023), and on the other side, small-cell carcinomas made of the typical small, round, blue cells positive for a neuroendocrine marker were observed. This tumor co-existence is a rare event. Although mouse lung neuroendocrine tumors have been genetically modeled by combined knockouts of different tumor suppressor genes (Hamad et al., 2022), studies indicate that small-cell neuroendocrine carcinomas do not develop spontaneously in mice (Janker et al., 2018). This observation may provide information about the carcinogenic nature of the tumor-derived CFFs.

Another interesting finding in breast tumor-treated mice is the presence of cells of putative epithelial origin and intensely expressing estrogen and progesterone receptors, CK7 and HER-2, in the subcapsular sinuses of mediastinal lymphoid nodes. This fact suggests that these lung adenocarcinoma conglomerates already contribute to a pool of circulating cells, which may be disseminating and colonizing. Dissemination and colonization are processes of the metastatic phenomenon that is considered a late event relative to tumor initiation (Hu and Curtis, 2020; Riggio et al., 2021). In the present study, as in the previous study (Berlanga-Acosta et al., 2022c), an astounding finding is that pre-malignant changes and malignant tumors may be elicited in a narrow temporary window, which is particularly challenging under the scope of the canonic multistage and long path of initiation–promotion–progression (Compton, 2020).

The *in vitro* experiment conducted in pre-hatched zebrafish embryos was an accurate and reliable alternative to confront the current observations accrued in nude mice. Zebrafish (*D. rerio*) is increasingly considered a valuable non-mammalian vertebrate to model cancer, given the evolutionary conservation of cancer-related programs between human and zebrafish (Hason and Bartunek, 2019). Our primary goal was examining whether exposing the embryos to the mammary carcinoma CFF during their intense organogenesis period translated to the expression of cancer markers as mature proteins, which would suggest that some signaler(s) contained in the pathologic CFF “interacted” with the embryo cells and perturbed the endogenous regulatory mechanisms of protein expression. This hypothesis is not only supported by the fact that human cancer markers were expressed by embryo cell populations but also because these embryos were morphologically and functionally impacted by the tumor-derived homogenates, as exemplified by persistent bradycardia. Since none of these disorders were ever found in embryos incubated with the healthy tissue-derived homogenate, the hypothesis of embryo cell-unspecific reactivity before the challenge of foreign mammal-derived components in the filtrate is, therefore, discarded. Further studies are warranted in this respect.

The fact that we have been unable so far to identify the tumor-derived signalers that may elicit the *in vivo* carcinogenic response is a major limitation of this study. Nevertheless, this study conclusively confirms that the CFF is a vector of some “malignant code” contained within human non-transmissible tumor cells, which may implement a carcinogenesis process. This study is also the fourth in a line that supports the hypothetical existence and transmissibility of a “pathologic cellular memory” encrypted in the “diseased cells” with no interspecies barrier that is able to impose the recapitulation of the human donor pathologic traits. In addition to the potential therapeutic significance derived from the identification of these carcinogenesis primers, we deem that these studies offer an additional practical and useful platform for *in vivo* cancer modeling.

## Data Availability

The original contributions presented in the study are included in the article/Supplementary Material; further inquiries can be directed to the corresponding author.

## Abbreviations

AAH, atypical adenomatous hyperplasia; CFF, cell-free filtrate (s); c-Myc, multifunctional transcription factor; CRP, C-reactive protein; CK-7, cytokeratin 7; DNA, deoxyribonucleic acid; H/E, hematoxylin/eosin; HER-2, human epidermal growth factor receptor 2; IDC, invasive ductal carcinoma; IHC, immunohistochemistry; IL-1, interleukin-1; IL-6, interleukin-6; MDA, malondialdehyde; PCNA, proliferating cell nuclear antigen; RNA, ribonucleic acid; TERT, telomerase reverse transcriptase; TNFα, tumor necrosis factor α; TTF-1, thyroid transcription factor 1.
